# Retrospective study comparing MR-guided radiation therapy (MRgRT) setup strategies for prostate treatment: repositioning vs. replanning

**DOI:** 10.1186/s13014-019-1349-2

**Published:** 2019-08-06

**Authors:** Jung-in Kim, Jong Min Park, Chang Heon Choi, Hyun Joon An, Yi-Jun Kim, Jin Ho Kim

**Affiliations:** 1 Department of Radiation Oncology, Seoul National University Hospital, Seoul, Republic of Korea; 2 Biomedical Research Institute, Seoul National University Hospital, Seoul, Republic of Korea; 3 Institute of Radiation Medicine, Seoul National University Medical Research Center, Seoul, Republic of Korea; 4 Center for Convergence Research on Robotics, Advanced Institutes of Convergence Technology, Suwon, Republic of Korea

**Keywords:** Soft-tissue repositioning, Adaptive replanning, Prostate radiotherapy, MRI-guided treatment

## Abstract

**Background:**

This study compared adaptive replanning and repositioning corrections based on soft-tissue matching for prostate cancer by using the magnetic resonance-guided radiation therapy (MRgRT) system.

**Methods:**

A total of 19 patients with prostate cancer were selected retrospectively. Weekly magnetic resonance image (MRI) scans were acquired for 5 weeks for each patient to observe the anatomic changes during the treatment course. Initial intensity-modulated radiation therapy (IMRT) plans (iIMRT) were generated for each patient with 13 coplanar ^60^Co beams on a ViewRay™ system. Two techniques were applied: patient repositioning and replanning. For patient repositioning, one plan was created: soft-tissue (prostate) matching (*Soft*). The dose distribution was calculated for each MRI with the beam delivery parameters from the initial IMRT plan. The replanning technique was used to generate the *Adaptive* plan, which was the reoptimized plan for the weekly MRI. The dose-volumetric parameters of the planning target volume (PTV), bladder, and rectum were calculated for all plans. During the treatment course, the PTV, bladder, and rectum were evaluated for changes in volume and the effect on dosimetric parameters. The differences between the dose-volumetric parameters of the plans were examined through the Wilcoxon test. The initial plan was used as a baseline to compare the differences.

**Results:**

The *Adaptive* plan showed better target coverage during the treatment period, but the change was not significant in the *Soft* plan. There were significant differences in D_98%_, D_95%_, and D_2%_ in PTV between the *Soft* and *Adaptive* plans (*p* < 0.05) except for D_mean_. There was no significant change in D_max_ and D_mean_ as the treatment progressed with all plans. All indices for the *Adaptive* plan stayed the same compared to those of iIMRT during the treatment course. There were significant differences in D_15%_, D_25%_, D_35%_, and D_50%_ in the bladder between the *Soft* and *Adaptive* plans. The *Adaptive* plan showed the worse dose sparing than the *Soft* plan for the bladder according to each dosimetric index. In contrast to the bladder, the *Adaptive* plan achieved better sparing than the *Soft* plan during the treatment course. The significant differences were only observed in D_15%_ and D_35%_ between the *Soft* and *Adaptive* plans (*p* < 0.05).

**Conclusions:**

Patient repositioning based on the target volume (*Soft* plan) can relatively retain the target coverage for patients and the OARs remain at a clinically tolerance level during the treatment course. The *Adaptive* plan did not clinically improve for the dose delivered to OARs, it kept the dose delivered to the target volume constant. However, the *Adaptive* plan is beneficial when the organ positions and volumes change considerable during treatment.

## Background

The goal of radiation therapy is to deliver an accurate prescription dose to the target while minimizing the dose to normal tissue. External beam radiotherapy (EBRT) is a common definitive treatment option for localized prostate cancer [1–7]. In the last decade, significant technological advances in EBRT have been achieved. Intensity-modulated radiation therapy (IMRT) is a type of conformal radiation therapy and is widely used to treat prostate cancer [1, 7, 8]. However, the complexity of the treatment delivery and inter- and intra-fraction variations are a concern when using IMRT for prostate cancer [9, 10]. Adding margins around the clinical target volume (CTV) can account for these variations to ensure target coverage. Gill et al. reported that when daily cone beam computed tomography (CBCT) was used for soft-tissue alignment of the prostate, a PTV margin of 3 mm allowed sufficient coverage for CTV [11]. On the other hand, in Engels’s study, margins of 6 mm LR and 10 mm AP and CC were used in patients who did not hve markers implanted in them. Margins of 3 mm LR and 5 mm AP and CC were used for patients with implanted markers [12]. However, expanding the irradiated volume consequently increases normal organ toxicity, which is limited by the tolerances of the bladder and rectum [8, 13]. In recent years, advances in image guidance have allowed for better localization of the prostate [14–20]. Using image guidance together with IMRT delivers the dose to the prostate more precisely with smaller margins to decrease the doses to the rectum and bladder. Image-guided radiation therapy (IGRT) technology has been widely adopted to provide anatomic information with the patient in the treatment position. With image guidance (typically X-ray imaging such as kilovolt (kV), megavolt (MV), and CBCT), the patient positions in the daily images are registered to the planning images based on either bony landmarks or soft tissues [16–18, 21, 22]. Furthermore, fiducial markers for prostate IGRT have been in use since the 1990s and O’Neill et al. reviewed the evidence for the use of fiducial markers in clinical practice [23]. Image guidance corrects not only for patient position variations but also for changes in the target volume and shapes. Adaptive radiation therapy (ART) has been introduced to compensate for variations in patient treatment during a radiotherapy course [24–26]. ART techniques can be categorized as offline or online adaptive planning [27]. Offline adaptive planning uses a feedback strategy for current treatment by incorporating the obtained daily treatment images. This adaptive planning reflects patient-specific anatomic variations and provides a reoptimized treatment plan for the remaining treatment fractions. Online adaptive planning is the optimal strategy for each fraction delivery because the treatment plan is reoptimized while the patient is waiting on the treatment table. However, clinical implementation of both offline and online adaptive techniques with a common imaging modality based on X-rays is still in the development phase because it is not sufficient to modify the treatment plan according to anatomic changes on inter- and intra-fraction bases. In 2009, Thongphiew et al. compared three online IGRT techniques (bony-anatomy matching, soft-tissue matching, and online replanning) for prostate IMRT treatment using daily CBCT [18]. They demonstrated that CBCT can feasibly be used for reoptimizing the treatment plan online, which has significant benefits when a high degree of deformation or differential organ position displacement occurs. Clinical implementation of ART for prostate cancer has been greatly promoted by image guidance with onboard CBCT [28–31]. Onboard CBCT allows the target volume and adjacent anatomies to be localized before treatment of a patient in the treatment position. However, CBCT has insufficient image quality compared to planning computed tomography (CT) to provide an accurate delineation of structures and dose calculation owing to the higher noise and lower contrast [29, 32]. Furthermore, the inconsistency between the CT number to electron density curves of CT and CBCT images can result in a discrepancy in the dose calculations.

Recently, the high soft-tissue contrast and real-time imaging capability of magnetic resonance imaging (MRI) have allowed for more accurate assessment of inter- and intra-fraction variations [33–36]. MR-guided radiotherapy (MRgRT) systems that integrate MR scanners with radiation delivery machines can potentially facilitate the online ART strategy. ViewRay™ (ViewRay Inc., Cleveland, OH, USA) is the first commercial MRgRT system and has been treating patients since 2014 [37]. Much work has been carried out on prostate treatment with the MRgRT system because it is very useful for identifying the prostate as well as detecting normal tissues [33, 34, 38–40]. The present study compared adaptive replanning and repositioning corrections based on soft-tissue matching for prostate cancer with the ViewRay system in a retrospective manner.

## Methods

### Patient data and study design

After institutional review board approval, a total of 19 patients with prostate cancer were selected for this study in a retrospective manner. Low-risk patients did not receive androgen deprivation therapy (ADT). Intermediate and high risk patients received neoadjuvant ADT. Table 1 summarizes the patients’ clinical characteristics. All patients were planned with IMRT using ViewRay. A CT image set and MRI image set of each patient in the supine position were acquired once before treatment. The Brilliance Big Bore™ CT simulator (Philips, Cleveland, OH, USA) was used for CT scanning with a slice thickness of 1.5 mm. Each patient was immobilized with Smart Rest™ (Chunsung, Seoul, Republic of Korea), which combines the knee fix and feet fix. The initial CT image (iCT) sets were only used to calculate the dose for the MRIdian™ system (ViewRay Inc., Cleveland, OH, USA); no CT images were taken during treatment. Initial MRI (iMRI) was used to draw the contours and the reference image. All patients underwent daily MRI scans for setup verification prior to treatment during the treatment period. Of these, only weekly MRI images were included in the study. All MRIs (six MRI sets per patient) were acquired on a 0.35 T ViewRay scanner system (ViewRay Inc., Cleveland, OH, USA). A non-contrast true fast imaging with steady-state precession (TRUFI) sequence was used for all MRI scanning [41]. The MRI resolution was 1.5× 1.5 × 3.0 mm^3^ with a typical imaging time of 25 s and a field of view of 54 × 47 × 43 cm^3^. The CTV was defined to include prostate and seminal vesicles. The planning target volume (PTV) was generated by adding margins of 3 mm and 5 mm in all directions to the prostate and seminal vesicles, respectively. The rectum and bladder were not controlled for filling. These structures (six RT structure sets per patient) were delineated on each MRI by a physician to ensure that the contouring practice was consistent. The prescription dose to the PTV was 70 Gy in 28 fractions for all patients. Two strategies for MRgRT plans were considered. One plan used the repositioning technique, which was called as the *Soft* plan. The repositioning technique finds the best matches of the target volume between the iMRI and weekly MRI (nMRI). Patient repositioning was performed by translational correction because of the motion limitations of the ViewRay couch. The other plan used the replanning technique for every nMRI, which was called as the *Adaptive* plan. Thus, each patient had one initial IMRT plan (iIMRT), five *Soft* plans, and five *Adaptive* plans.

**Table 1:** Summary of patients’ clinical characteristics

Age (years)	Mean (range)	77 (65–86)
T stage	T1	2 (10.5%)
	T2	11 (57.9%)
	T3	2 (10.5)
	T4	4 (21.1%)
GS score	6 (3 + 3)	3 (15.8%)
	7 (3 + 4)	3 (15.8%)
	7 (4 + 3)	5 (26.3%)
	8 (4 + 4)	7 (36.8%)
	9 (4 + 5)	1 (5.3%)
^a^PSA	Median (range)	18.89 (2.19–161.95)
	Mean (SD)	27.78 (35.63)
^b^ADT use	10 (52.63%)	

^a^*PSA* prostate-specific antigen; ^b^*ADT* androgen deprivation therapy

### Techniques and treatment plans

The iCT was deformed to iMRI to generate the deformed CT (dCT) for dose calculation. The initial structure set (iRS) contoured on iMRI was also included in the treatment planning. A total of 13 fields (five beam groups) were used to generate an iIMRT plan for each patient with the tri-^60^Co system. The gantry angles of the fields were 0° (group 1); 24°, 144°, and 264° (group 2); 48°, 168°, and 267° (group 3); 72°, 192°, and 312° (group 4); and 96°, 216°, and 336° (group 5). The values for the IMRT efficiency that smoothed the fluence map intensity pattern (could be set from 0 to 1) and maximized the deliverable beam-on segments per field were 0.5 and 10, respectively. The optimization and dose calculation were performed with an imaging surface coil in the presence of a magnetic field and using a calculation grid of 3 mm. The Monte Carlo calculation algorithm developed by the manufacturer (ViewRay Inc., Cleveland, OH) was used to calculate the dose. The number of histories in the system was set to 2.4 × 10^6^. This stetting achieves about 1% statistical uncertainty for a dose grid of 3 mm cubed voxels. The optimization was performed according to National Comprehensive Cancer Network (NCCN) guidelines, and each plan was normalized to cover 100% of the PTV with 95% of the prescription dose. The MRIdian™ system use a convex nonlinear programing model for dose optimization. Table 2 shows the dose optimization parameters for the target and OARs. For a relatively fair comparison, all plans used these optimization parameters. Finally, the iIMRT plan was determined according to several beam delivery parameters. For each patient, six sets of MRIs (nMRI) and structure sets (nRS) were scanned during the treatment course and used in this study. For the *Soft* plan, the isocenter from iIMRT was relocated to the center of the PTV from nRS with nMRI. After the patient was repositioned based on target volume, the dose (from the iIMRT) was recalculated on nMRI with nRS. In contrast, the *Adaptive* plan was generated from the *Soft* plan with reoptimization by using the same constraints and associated weights for iIMRT. The d’CT was generated by deforming dCT to nMRI and used to calculate the dose for each plan. Each plan was compared at a prescription dose of 70 Gy.

**Table 2:** Summary of MRIdian™ optimization parameters for target volume and OARs

Structure	Importance		Power		Threshold (Gy)	Prescription (Gy)	Offset
	Lower	Upper	Lower	Upper			
Skin	–	1	–	1	21	–	
Rectum	–	1	–	1	56	–	
Bladder	–	1	–	1	56	–	
^a^PTV	2	2	2	2	–	70	0.5

^a^*PTV* planning target volume

### Dose-volumetric evaluation and comparison

The dose-volumetric parameters of the PTV, bladder, and rectum were calculated for all plans. The dosimetric index D_x%_ is defined as the dose to x% of volume of a structure. For the PTV, the mean dose (D_mean_), D_95%_, D_98%_, and D_2%_ were calculated. The conformity index (CI), homogeneity index (HI), and gradient index (GI) were calculated as follows [42, 43]:

$$ Conformity\ index\ (CI)=\frac{Volume\ of\ reference\ isodose}{Volume\ of\ target\ volume} $$

$$ Homogeneity\ index\ (HI)=\frac{D_{2\%}-{D}_{98\%}}{D_{50\%}} $$

$$ Gradient\ index\ (GI)=\frac{V_{50\%} of\ the\ prescription\ dose}{V_{100\%}\  of\ the\ prescription\ dose} $$

For the bladder and rectum, D_15%_, D_25%_, D_35%_, and D_50%_ were calculated. During the treatment course, the PTV, bladder, and rectum were evaluated for changes in volume and their effect on the dosimetric parameters. Each technique was compared according to the abovementioned dose-volumetric parameters. Wilcoxon tests were performed at the 95% confidence level to examine the statistical significance of differences between the dose-volumetric parameters of the plans. The iIMRT (i.e., IMRT plan for the first fraction) was used as a criterion for comparing the differences. The given plan was defined as “reproducible” for both PTV and OARs, respectively, if the difference was less than 5% compared to the iIMRT.

## Results

### Dose-volumetric evaluation and comparison of PTVs

Table 3 lists the results of dose-volumetric evaluation and a comparison of the PTVs for each plan. There were significant differences in D_98%_, D_95%_, and D_2%_ in PTV between the *Soft* and *Adaptive* plans (*p* < 0.05), except for D_mean_. For each dosimetric index, one plan group including the iIMRT exhibited an average value relative to the prescription dose and its standard deviation (Mean ± SD). With regard to the index related to target coverage, the *Adaptive* plan clearly demonstrated superior results for D_98%_ (99.1 ± 0.3%). For D_95%_, the average value for *Adaptive* plan was always 100% because each plan was normalized to cover 100% of the PTV with 95% of the prescription dose. The difference was statistically significant compared with that of the *Soft* plan (*p* = 0.011). Compared with iIMRT, the average difference for the *Adaptive* plan was less than 1% for all dose-volumetric evaluations. The percentage of reproducible plans for D_98%_ was 77.9% with the *Soft* plan.

**Table 3:** Dose-volumetric evaluation and comparison between PTVs during the treatment

Indices	Plan	Mean ± SD (%)	*p*-value	% of reproducible plan^a^	Average difference (%)
D_mean_	*Soft*	102.5 ± 0.6	0.099	100	0.1 ± 0.9
	*Adaptive*	102.2 ± 0.3		100	−0.1 ± 0.5
D_98%_	*Soft*	96.6 ± 3.3	0.005*	77.9	−2.8 ± 4.1
	*Adaptive*	99.1 ± 0.3		100	−0.2 ± 0.6
D_95%_	*Soft*	98.5 ± 2.6	0.011*	92.6	−1.8 ± 3.2
	*Adaptive*	100		100	0
D_2%_	*Soft*	105.5 ± 1.0	< 0.001*	100	0.7 ± 1.0
	*Adaptive*	104.6 ± 0.4		100	−0.4 ± 1.0

*Statistically significant comparisons; ^a^ Percentage of plans with a difference of < 5% compared to the initial plan

**Fig. 1:**
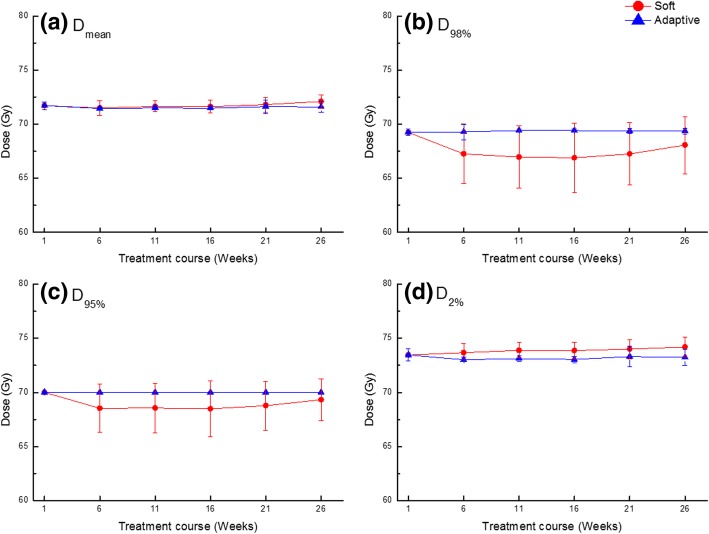
Averaged dose-volumetric values of (**a**) D_mean_, (**b**) D_98%_, (**c**) D_95%_, and (**d**) D_2%_ for the target volume during the treatment course

**Fig. 2:**
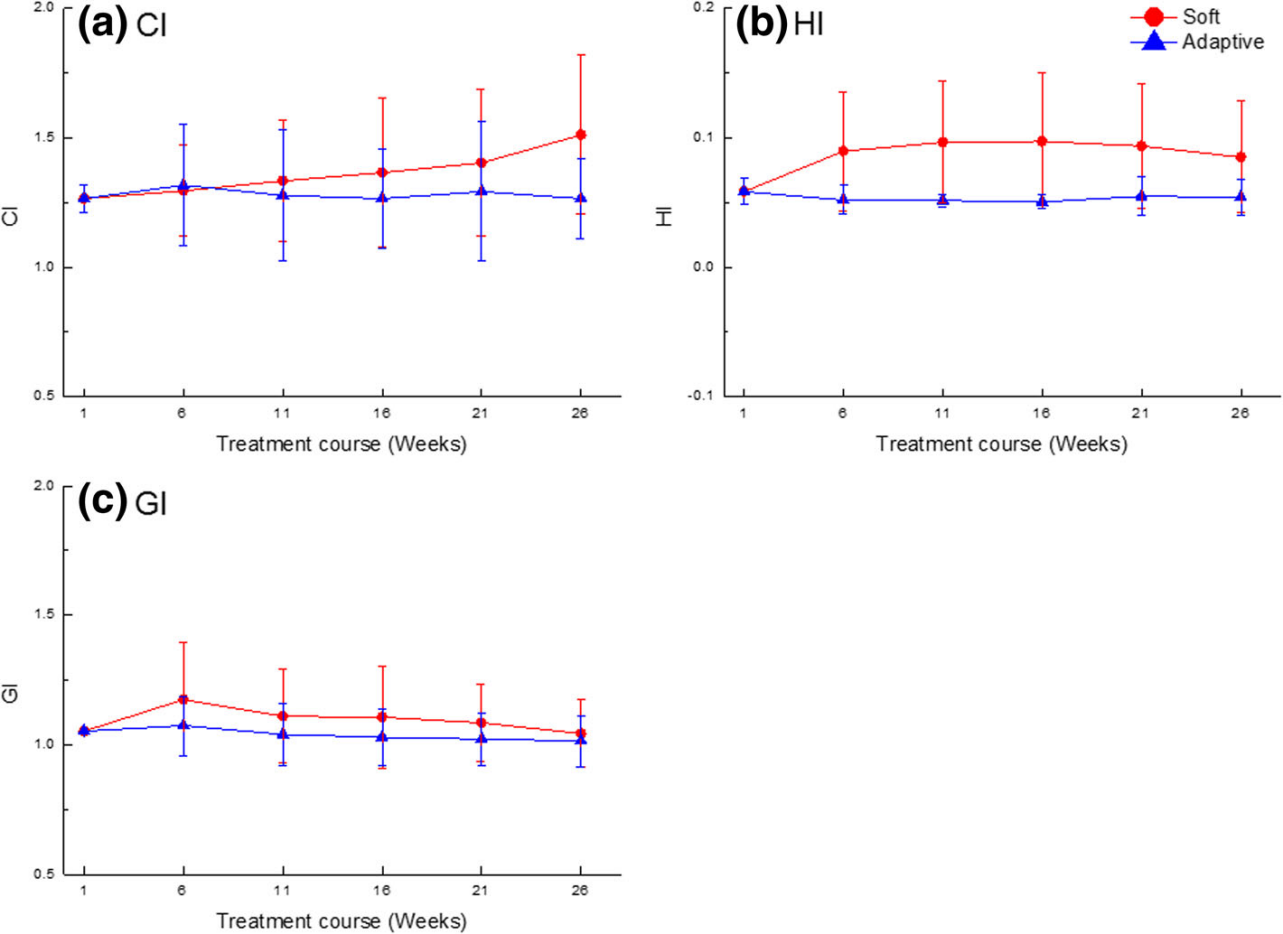
Averaged dosimetric indices of (**a**) CI, (**b**) HI, and (**c**) GI for the target volume during the treatment course

Figure 1 shows the averaged dose-volumetric values of the PTV for each plan and all patients during treatment. The target coverage during the treatment course did change significantly with the *Soft* plan. There was no significant change in D_max_ and D_mean_ as the treatment progressed with all plans. Figure 2 shows the averaged CI, HI, and GI of the PTV for all plans during treatment. The average CI values during the treatment course were 1.38 ± 0.27 with the *Soft* plan, and significant changes were observed during the treatment course. In this analysis, the iIMRT was excluded because this was the reference plan. The CI value was lower in the *Adaptive* plan but not statistically significant compared to that in the *Soft* plans (*p* = 0.08). However, HI and GI values were also lower in the *Adaptive* plan and statistically significant, compared to the *Soft* plans (*p* < 0.05). The averaged HI and GI values for the *Soft* plan compared to those of iIMRT were 0.09 ± 0.05 and 1.11 ± 0.18, respectively. All indices for the *Adaptive* plan stayed the same compared to those of iIMRT during the treatment course; the average values of CI, HI, and GI were 1.28 ± 0.22, 0.06 ± 0.01, and 1.04 ± 0.11, respectively*.*

### Dose-volumetric evaluation and comparison for the bladder

**Fig. 3:**
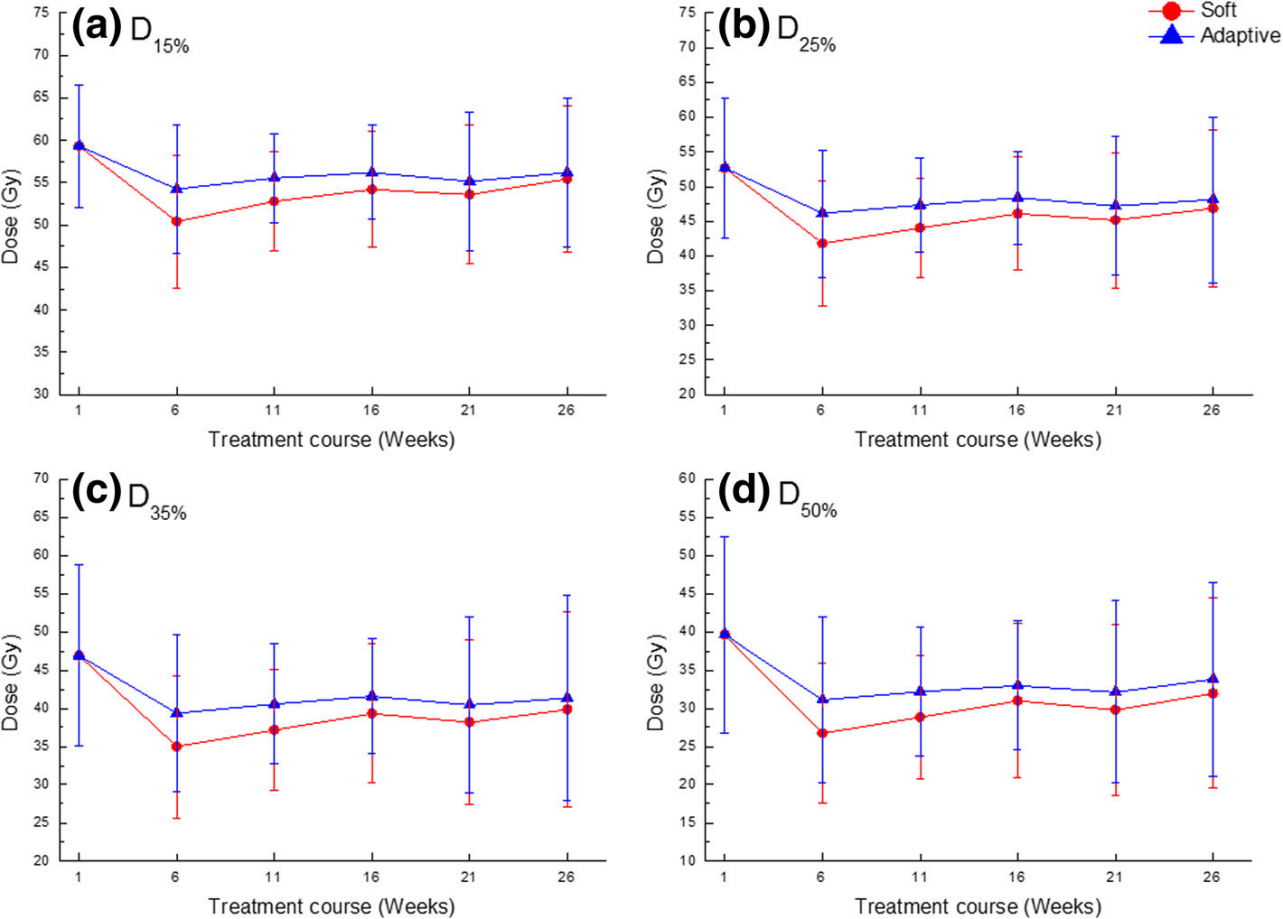
Averaged dose-volumetric values of (**a**) D_15%_, (**b**) D_25%_, (**c**) D_35%_, and (**d**) D_50%_ for the bladder during the treatment course

Figure 3 shows the averaged dose-volumetric values of the bladder for each technique and all patients during treatment. With the *Soft* and *Adaptive* plans, the average D_15%_ values excluding that of iIMRT during the treatment course were 53.3 ± 7.6 and 55.5 ± 7.1 Gy, respectively. The average D_25%_ values were 44.8 ± 9.2 and 47.4 ± 9.0 Gy, respectively. The average D_35%_ values were 37.9 ± 10.1 and 40.7 ± 10.2 Gy, respectively. The average D_50%_ values were 29.7 ± 10.3 and 32.5 ± 10.4 Gy, respectively. The *Adaptive* plan did not achieve a better result than the that using the *Soft* plan during the treatment course. However, all plans had similar values for the standard deviation and exhibited a tolerable dose distribution of the bladder. Table 4 lists the results of the dose-volumetric evaluation and compares the plans for the bladder. There were significant differences in D_15%_, D_25%_, D_35%_, and D_50%_ in the bladder between the *Soft* and the *Adaptive* plans. For each dosimetric index, one plan group including the iIMRT showed the average value relative to the prescription dose and its standard deviation (Mean ± SD). The *Adaptive* plan showed the worse dose sparing compared with the *Soft* plan for bladder according to each dosimetric index. The difference was statistically significant compared to that for the *Soft* plan (*p* < 0.05). Even though the *Adaptive* plan showed a higher dose in the bladder than the *Soft* plan, the average percentage of differences compared to iIMRT showed lower values than those of the *Soft* plan. However, the percentages of reproducible plans for D_15%_, D_25%_, and D_35%_ were 35.8, 28.4, and 25.3%, respectively, which were higher values than those of the *Soft* plan except for D_50%_.

**Table 4:** Dose-volumetric evaluation and comparison of plans for the bladder during the treatment course

Indices	Plan	Mean ± SD (%)	*p*-value	% of reproducible plan^a^	Average difference (%)
D_15%_	*Soft*	77.6 ± 7.8	0.01*	33.7	−9.14 ± 15.1
	*Adaptive*	80.2 ± 8.2		35.8	− 5.4 ± 14.8
D_25%_	*Soft*	65.9 ± 9.8	0.01*	25.3	−12.5 ± 23.5
	*Adaptive*	69.0 ± 10.6		28.4	−7.2 ± 24.9
D_35%_	*Soft*	56.3 ± 11.2	0.016*	23.2	−14.6 ± 33.5
	*Adaptive*	59.6 ± 12.2		25.3	−8.0 ± 37.4
D_50%_	*Soft*	44.8 ± 11.5	0.02*	23.2	−16.4 ± 48.5
	*Adaptive*	48.1 ± 12.7		20.0	−8.0 ± 56.3

*Statistically significant comparisons; ^a^ Percentage of plans with a difference of < 5% compared to the initial plan

### Dose-volumetric evaluation and comparison for the rectum

**Fig. 4:**
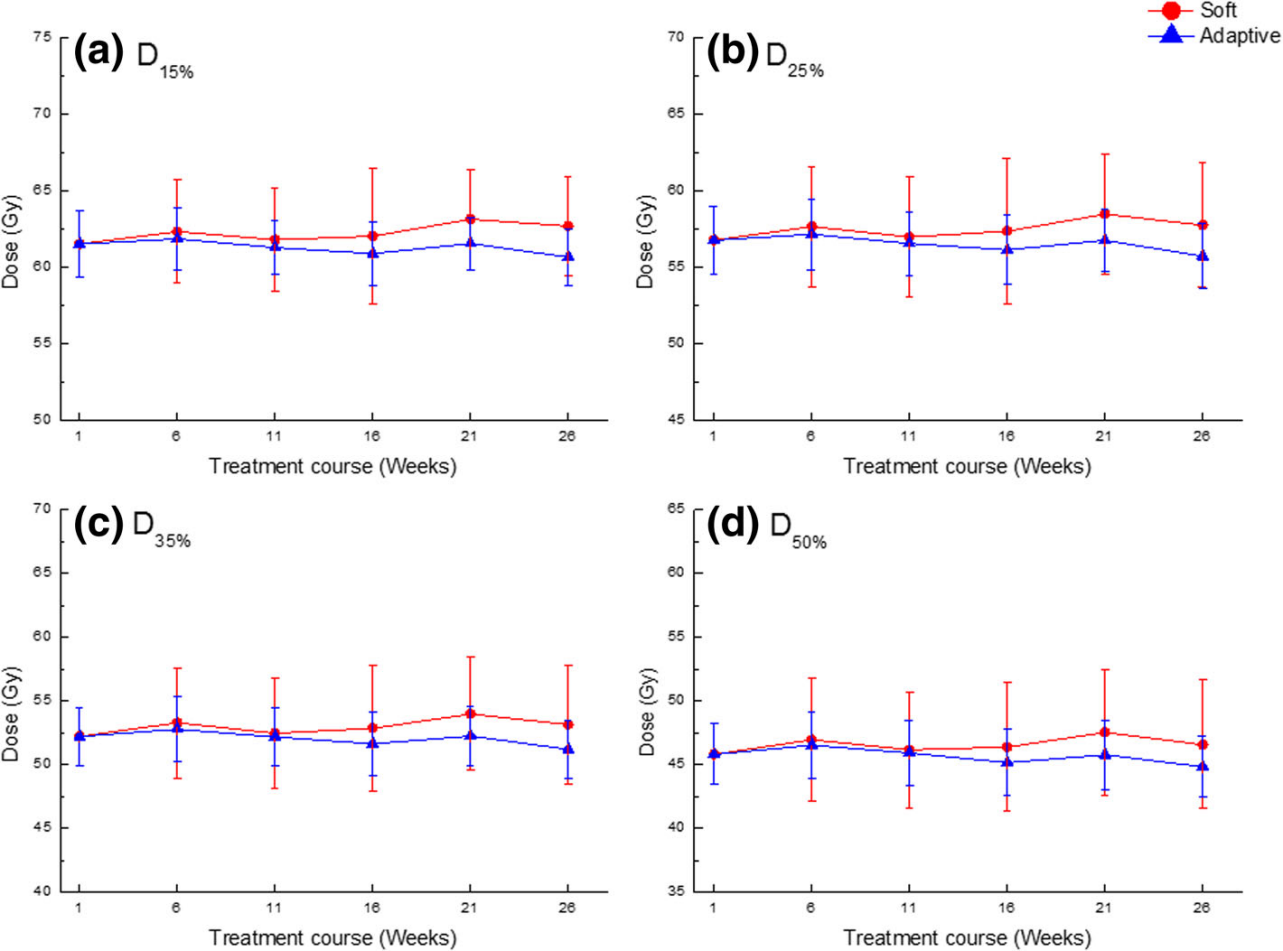
Averaged dose-volumetric values of (**a**) D_15%_, (**b**) D_25%_, (**c**) D_35%_, and (**d**) D_50%_ for the rectum during the treatment course

Figure 4 shows the averaged dose-volumetric values of the rectum for each technique and all patients during treatment. With the *Soft* and *Adaptive* plans, the average D_15%_ values excluding that of iIMRT during the treatment course were 62.4 ± 3.5 and 61.3 ± 1.9 Gy. The average D_25%_ values were 57.7 ± 4.1 and 56.5 ± 2.2 Gy; the average D_35%_ values were 53.2 ± 4.5 and 52.0 ± 5.4 Gy; and the average D_50%_ values were 46.7 ± 4.8 and 45.6 ± 2.6 Gy. All plans were below the tolerance dose of rectum. In contrast to the bladder, the *Adaptive* plan achieved better sparing than that by the *Soft* plan during the treatment course and showed the smallest standard deviation. Table 5 lists the results of the dose-volumetric evaluation and compares the plans for the rectum. The differences were only observed in D_15%_ and D_35%_ between the *Soft* and *Adaptive* plans, statistically (*p* < 0.05). For each dosimetric index, one plan group including iIMRT showed the average value relative to the prescription dose and its standard deviation (Mean ± SD). The *Adaptive* plan shows less average value and lower standard deviation for the rectum than the *Soft* plan according to each dosimetric index. However, no differences were observed between the plans in D_25%_ and D_50%_ (*p* > 0.05). With the *Soft* plan, the percentages of reproducible plans for D_15%_, D_25%_, D_35%_ and D_50%_ were 87.4, 87.4, 87.4, and 86.3%, respectively. The average percentages of difference compared to iIMRT had positive mean values and large standard deviations: 1.1 ± 6.4%, 1.1 ± 7.7%, 1.2 ± 8.8%, and 1.2 ± 9.9% for D_15%_, D_25%_, D_35%_ and D_50%_, respectively. With the *Adaptive* plan, on the other hand, the percentages of reproducible plans for D_15%_, D_25%_, D_35%_ and D_50%_ were 89.5, 86.3, 81.1, and 76.8%, respectively. The average percentages of difference compared to iIMRT had negative mean values and small standard deviations: − 0.8 ± 3.0%, − 1.0 ± 3.7%, − 1.0 ± 4.4%, and − 1.1 ± 5.8% for D_15%_, D_25%_, D_35%_ and D_50%_, respectively.

**Table 5:** Dose-volumetric evaluation and comparison of plans for the rectum during the treatment course

Indices	Plan	Mean ± SD (%)	*p*-value	% of reproducible plan^a^	Average difference (%)
D_15%_	*Soft*	62.3 ± 2.8	0.009*	87.4	1.1 ± 6.4
	*Adaptive*	61.3 ± 1.5		89.5	−0.8 ± 3.0
D_25%_	*Soft*	57.5 ± 3.2	0.16	87.4	1.1 ± 7.7
	*Adaptive*	56.5 ± 1.8		86.3	−1.0 ± 3.7
D_35%_	*Soft*	75.7 ± 5.1	0.033*	87.4	1.2 ± 8.8
	*Adaptive*	74.4 ± 2.7		81.1	−1.0 ± 4.4
D_50%_	*Soft*	66.5 ± 5.6	0.059	86.3	1.2 ± 9.9
	*Adaptive*	65.2 ± 2.9		76.8	−1.1 ± 5.8

*Statistically significant comparisons; ^a^ Percentage of plans with a difference of < 5% compared to the initial plan

## Discussion

**Fig. 5:**
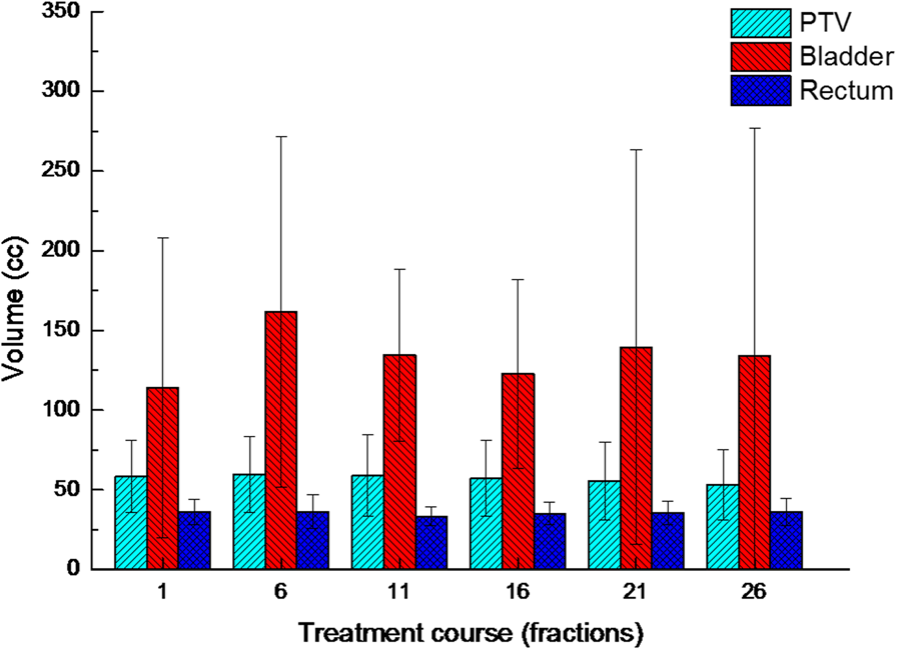
Average volume variation of organs for all patients during the treatment course

**Fig. 6:**
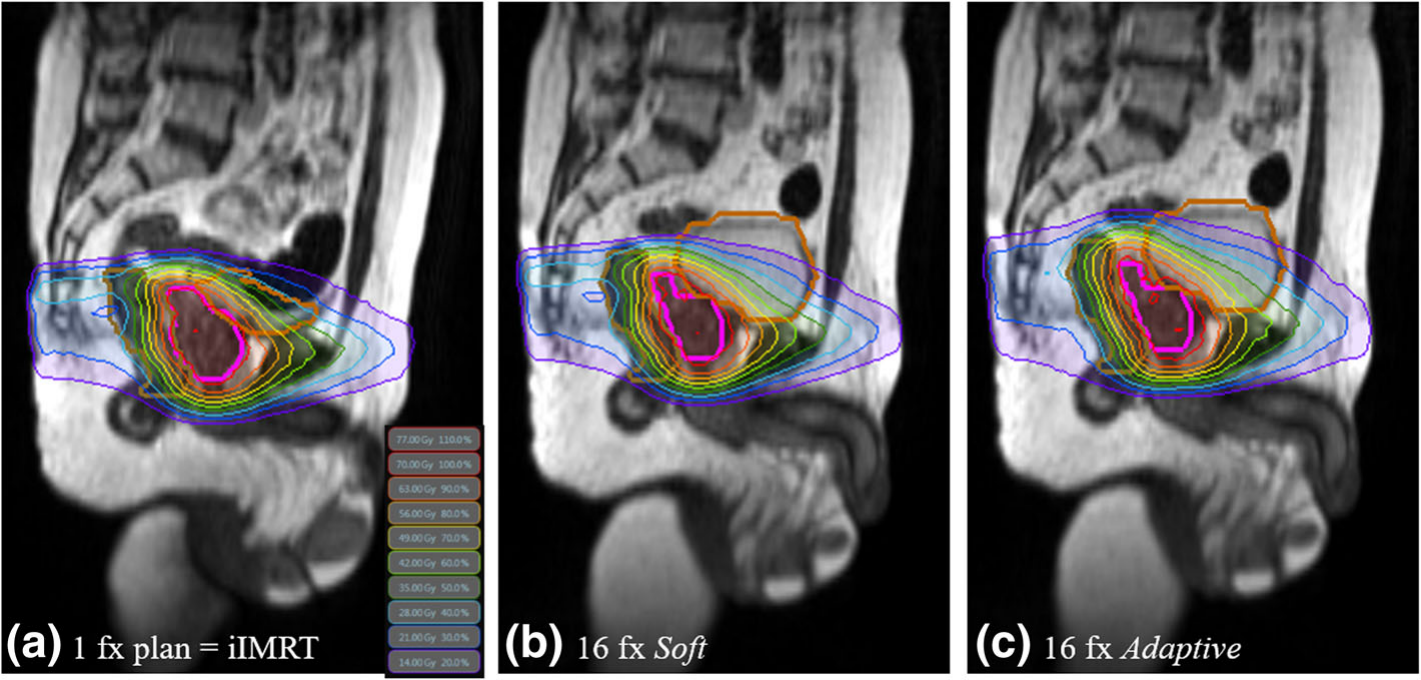
Representative dose distributions with three plans: (**a**) initial, (**b**) *Soft*, and (**c**) *Adaptive*. All plans excluding the initial plan had the same organ structures and MRI

This study explored different MRgRT techniques to compensate for inter-fraction errors during prostate treatment. The *Soft* plan, which repositions the patient by using the target volume, may be sufficient for correcting anatomic variations. For OARs with the *Soft* plan, the dose to the bladder was relatively low, while the dose to the rectum was relatively consistent during treatment. The *Adaptive* plan, which is a replanning technique, may be suitable for correcting anatomic variations, and the doses to the OARs remained relatively consistent during treatment. The *Adaptive* plan performed very similarly to the initial plan and compensated for inter-fraction uncertainty. The large variations in the mean values and standard deviation indicate that substantial fluctuations in target coverage can occur weekly with IGRT techniques based on patient repositioning. Only the replanning technique (i.e., *Adaptive* plan) maintained a consistent quality during the treatment period in terms of target coverage. However, the *Adaptive* plan did not achieve the better results in terms of sparing the bladder during the treatment course. In this study, the enrolled patients were not controlled for bladder and rectum filling because they had been treated with gated radiotherapy using the ViewRay system. Thus, the bladder and rectum were filled with various volumes during the treatment course. Figure 5 shows the averaged volume variation for all patients during the treatment course. The changes in the bladder volume were significant with large standard deviations. Significant variations in the bladder volume affect both the bladder dose volume and positions of adjacent organs [44–46]. The *Adaptive* plan did achieve the better results for rectum sparing because the variation in the rectum volume was not significant. Figure 6 compares the example plans. In this case, the *Soft* plan had better OAR sparing than the *Adaptive* plan while missing target coverage. Thongphiew et al. used daily CBCT and also reported that the adaptive technique does not achieve the better OAR sparing for all fractions [18]. They discussed the limitations of CBCT. Compared to CT, CBCT images make it more difficult to distinguish the boundaries of soft-tissue organs (e.g., the prostate, bladder, and rectum) because of more noise and poorer soft-tissue contrast. In this study, however, a 0.35 T MRI was used with high soft-tissue contrast and real-time imaging capability to exactly define the soft-tissue organ variations. Even low-field-strength MRI gives improved anatomic visualization compared with CT and CBCT [47]. The MRI allowed for the most accurate delineation of soft tissues and observation of organs changing. Thus, we adopted PTV margins of 3 mm and 5 mm in all directions for the prostate and seminal vesicles, respectively. McPartlin et al. reported that the CTV-to-PTV margin can be safely reduced to less than 3 mm in MRI-guided prostate adaptive radiotherapy because of high soft-tissue contrast and real-time imaging capability [48]. On the other hand, the plan quality was not better than that of a treatment-based linear accelerator (linac) because of the large penumbra of the ^60^Co source and a large width of the multileaf collimator (MLC). The patient positioning techniques were also limited because only translational corrections were available. An MR-linac with 6 MV beam is available in a clinic, and this system provides a better plan quality than a system equipped with ^60^Co beams because of small penumbra and a small width of MLC [49, 50]. In addition, adaptive planning will increase the time between the imaging and the beam delivery. The intra-fractional motion and deformation also increase with time [51]. These could be handled by using the gating delivery technique in treatment delivery. Furthermore, the *Adaptive* plan is not the actual dose delivered and the actual delivered dose should be assessed in parallel after treatment.

## Conclusions

In this study, the MRgRT technique using adaptive planning was compared to the technique based on patient repositioning for 19 prostate cancer patient with the MRI. Patient repositioning based on the target volume (*Soft* plan) can relatively retain the target coverage for patients and the OARs remain at a clinically tolerance level during the treatment course. Although the *Adaptive* plan did not clinically improve for the dose delivered to OARs, it kept the dose delivered to the target volume constant. However, the *Adaptive* plan is beneficial when organ positions and volumes change considerably during treatment.

## Data Availability

Data sharing not applicable to this article because no datasets were generated or analyzed during the current study.

## Abbreviations

ADT: Androgen deprivation therapy
ART: Adaptive radiotherapy
CBCT: Cone-beam computed tomography
CI: Conformity index
CT: Computed tomography
CTV: Clinical target volume
dCT: Deformed CT
EBRT: External beam radiotherapy
GI: Gradient index
HI: Homogeneity index
iCT: Initial CT image
IGRT: Image-guided radiotherapy
iIMRT: Initial intensity-modulated radiation therapy plans
iMRI: Initial MRI
IMRT: Intensity-modulated radiation therapy
iRS: Initial structure set
kV: Kilovolt
Linac: Linear accelerator
MLC: Multileaf collimator
MRgRT: Magnetic resonance-guided radiation therapy
MRI: Magnetic resonance imaging
MV: Megavolt
NCCN: National comprehensive cancer network
nMRI: Weekly MRI
nRS: Weekly structure set
OARs: Organs at risk
PTV: Planning target volume
TRUFI: True fast imaging with steady-state precession
